# Metabolic Consequences of the Water We Drink: A Study Based on Field Evidence and Animal Model Experimentation

**DOI:** 10.3390/toxics11040315

**Published:** 2023-03-28

**Authors:** Janaína Caroline Wolfart, João Lucas Theodoro, Fernanda Coleraus Silva, Cíntia Mara Ribas de Oliveira, Nuno G. C. Ferreira, Ana Tereza Bittencourt Guimarães

**Affiliations:** 1 Laboratory of Biological Investigations, Universidade Estadual do Oeste do Paraná, Rua Universitária, Cascavel 2069, Paraná, Brazil; janainawolfart1@gmail.com (J.C.W.);; 2 Graduate Program in Biosciences and Health, Universidade Estadual do Oeste do Paraná, Rua Universitária, Cascavel 2069, Paraná, Brazil; 3 Graduate Program in Environmental Management, Universidade Positivo, Rua Professor Parigot de Souza, Curitiba 5300, Paraná, Brazil; 4 CIIMAR: Interdisciplinary Centre of Marine and Environmental Research, Universidade do Porto, Terminal de Cruzeiros de Leixões. Av. General Norton de Matos s/n, 4450-208 Matosinhos, Portugal; 5 School of Biosciences, Cardiff University, Museum Avenue, Cardiff CF10 3AX, UK

**Keywords:** banned pesticides, residual concentrations, chronic exposure, persistent pesticides, one health

## Abstract

The effect of the chronic consumption of water contaminated with residual concentrations of DDT’s metabolites (DDD—dichlorodiphenyldichloroethane and DDE—dichlorodiphenyldichloroethylene) found in the environment were evaluated on the biometric, hematological and antioxidant system parameters of the hepatic, muscular, renal and nervous tissues of Wistar rats. The results showed that the studied concentrations (0.002 mg.L^−1^ of DDD plus 0.005 mg.L^−1^ of DDE) could not cause significant changes in the hematological parameters. However, the tissues showed significant alteration in the activity of the antioxidant system represented by the increase in the activity of the enzymes gluthathione S-transferases in the liver, superoxide dismutase in the kidney, gluthathione peroxidase in the brain, and several changes in enzymatic activity in muscle (SOD, GPx and LPO). The enzymes alanine aminotransaminase (ALT) and aspartate aminotransaminase (AST) were also evaluated for the amino acids’ metabolism in the liver, with ALT showing a significant increase in the exposed animals. In the integrative analysis of biomarkers (Permanova and PCOA), the studied concentrations showed possible metabolic changes and damage to cellular structures evidenced by increased oxidative stress and body weight gain among the treated animals. This study highlights the need for further studies on the impact of banned pesticides still present in soils that may induce adverse effects in organisms that may prevail in future generations and the environment.

## 1. Introduction

Agricultural practices based on the intense use of pesticides, especially in areas near springs, are of most concern mainly due to the possible transfer of pesticides and contamination of the spring water [1,2,3,4]. Water can be considered an integrator of the biogeochemical processes that occur in a region [5], and any eventual contamination can cause losses to ecosystems and harm to human health, which can be more dangerous if these resources are used for human consumption [6]. As a result, particular attention should be paid to the water consumption from artesian wells resulting from the surface water table. These groundwaters may be contaminated with pesticides and consumed without any treatment [7].

Pesticides can reach groundwater through the percolation process, whose particles can be transported in a vertical direction, reaching specific depths and posing a potential groundwater contamination source [8]. As groundwater is considered a recalcitrant environment [9], pesticides tend to accumulate in it. This is becoming a serious public health issue, as 52% of Brazilian municipalities are supplied with underground water according to the TrataBrasil Institute [10]. As an example, in Brazil, there are more than 2.5 million artesian wells, of which 88% are illegal [11].

A study conducted in Brazil from 2014 to 2017 showed that in more than 2300 municipalities, the water consumed by the local population was contaminated by at least 27 different types of pesticides [11]. This study includes cities in the west of the state of Paraná, a region with strong agricultural activity and high pesticide use [11]. Between 2013 and 2018, an example from this region, a small-sized municipality [12] consumed, on average, approximately 40 kg of pesticide per inhabitant per year [13]. Owing to this high consumption of pesticides, the concentrations of pesticides used in the present study was based on data from this region. Here, 11 pesticides were detected [11], including the pesticide DDT (dichlorodiphenyltrichloroethane), its derivatives DDD (dichlorodiphenyldichloroethane) and DDE (diclhorodiphenyldichloroethylene)—pesticides that have been permanently banned since 2009 [14]. According to Faroon et al. (2019) [15], DDT and its metabolites are responsible for several harmful effects, which include hepatotoxic and hepatocarcinogenesis [16,17], neurological and neurodevelopment effects [18,19], reproductive and development effects [20,21], obesogenic effect [22,23,24,25], and suppression/stimulation of different immune responses [26,27].

The western Parana region mainly comprises soils classified as latosol, with mineralogical characteristics that promote a prolonged percolation process due to its fine granulometry and positive asymmetry. DDT attaches strongly to the soil and slowly decomposes into DDE and DDD, with a 2–15 year half-life in the soil [28,29]. Thus, a study with DDT residues becomes relevant, considering that DDD has an estimated half-life in water of up to 190 years [30] and DDE is the product of most DDT degradation reactions that occur in the environment [31]. Although the effects of exposure to high concentrations of DDD and DDE are well understood [15], the chronic effects of the trace concentration consumption of DDT are still poorly explored, especially considering the potential exposure of the population during their childhood, puberty and adulthood.

The present study aims to evaluate the chronic effects of consuming water with trace concentrations of the organochlorines DDD and DDE on the antioxidant system of muscle, liver, kidney and nervous tissues of Wistar rats in order to simulate the metabolic consequences of long-term consumption of contaminated water.

## 2. Materials and Methods

### 2.1. DDD and DDE

The selection of concentrations for this study was based on environmentally relevant concentrations. The municipality of Santa Tereza do Oeste is located in western Parana and has a history of widespread use of pesticides. In this municipality, soil collections were carried out at 12 urban and 12 georeferenced rural points, which showed the presence of DDT and its metabolites DDD and DDE [32]. The pesticide DDT ranged up to 0.019 mg.kg^−1^ soil, and its metabolites, DDD and DDE, ranged, respectively, up to 0.003 mg.kg^−1^ soil, and up to 0.012 mg.kg^−1^ soil. Due to their slow degradation, these DDT metabolites can remain in the environment for up to 190 years [30,31,33]. It is expected that they will reach the groundwater in similar concentrations.

In order to test the effects of the metabolites, DDD (4,4′-DDD Sigma-Aldrich Pestanal^®^, CAS Number: 72-54-8) and DDE (4,4′-DDE Sigma-Aldrich Pestanal, CAS Number: 72-55-9) were dissolved in absolute alcohol [34] in order to obtain a stock solution (0.04 mg.mL^−1^). The stock solution was stored at 4 °C (protected from light). Daily, the solution was diluted in water to reach a final concentration of 0.002 mg.L^−1^ of DDD and 0.005 mg.L^−1^ of DDE. These tested concentrations were based on the median concentration of DDD and DDE found in urban soil samples collected from the small city of Santa Teresa do Oeste (Parana, Brazil; Guimarães et al. unpublished data). Owing to the low degradation rates of these metabolites and the shallow water table in the area, the tested concentrations may be likely found in the water that the populations collect from artesian wells and also reach other organisms. The solution was offered to animals of the exposure group during the experimental period and aimed to simulate the synergic effect of chronic exposure to DDT residues, using water as a vehicle of contamination.

### 2.2. Experimental Design

Wistar male rats from the Central Animal Facility of Unioeste (State University of Western Paraná, Cascavel, Parana, Brazil) and all procedures developed in this study were approved by the Ethics Committee on Animal Use of Unioeste (Protocol n° 74-19).

**Figure 1:**
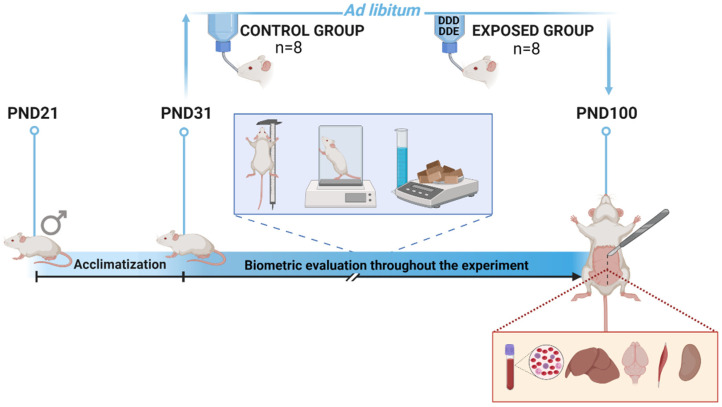
Experimental design used in this study. After 10 acclimation days (PND31), the control group received uncontaminated water until the end of the experiment (PND100) and the exposure group received water contaminated with DDD (0.002 mg.L^−1^) + DDE (0.005 mg.L^−1^) for 70 continuous days. Biometrics were evaluated for both groups between PND31 and PND100. Daily water consumption and weekly consumption of the standard diet were determined. Body weight and naso-anal length were measured weekly. (Figure created using BioRender.com).

At weaning on twenty-one post-natal days (PND21), animals were transferred to the Animal House of the Laboratory of Endocrine Physiology and Metabolism and randomly separated into two groups: control group (*n* = 8) and exposure group (*n* = 8), with each animal corresponding to a replicate (Figure 1). Rats were housed in polypropylene boxes at 22 ± 2 °C in a 12 h light/dark cycle with air exhaust. A standard diet for rodents (Nuvilab^®^, Nuvital Ltd.a, Parana, Brazil) was provided *ad libitum*. For nine days, the animals underwent an acclimatisation period when all received drinking water. On the 10th day (PND31), animals of the exposure group were provided with DDD + DDE contaminated water for 70 continuous days. Water was supplied *ad libitum* throughout the experiment.

### 2.3. Biometric Evaluation

Daily water consumption was determined by calculating the consumption difference between consecutive days, thus allowing the determination of the total ingested pesticide concentration. The weekly intake was determined for both groups by the difference between the food quota offered (300 g per week) and the subsequent week’s rejection. Total food consumption (TC) was determined at the end of the experiment by adding the daily food consumption.

Body weight (g) was measured using an analytic scale, and the naso-anal length (cm) was measured with each animal immobilised on a flat surface. The body weight and naso-anal length were evaluated weekly. The Lee index (IL—Equation (1)), total body weight gain (TMG—Equation (2)) and specific rate of body weight gain (Equation (3)) were calculated on a weekly basis:

(1) $$IL=\frac{\sqrt[{3}]{weight}\;(g)}{length\;(cm)}\;\times \;1000$$

(2) $$TMG=Final\;Weight\;\left(g\right)-Initial\;Weight\;(g)$$

(3) $$SRWG=\frac{Body\;weight\;gained\;during\;a\;specific\;period\;(dM)}{Initial\;body\;weight\;(Mdt)}$$

The feed efficiency was determined by means of the feed efficiency coefficient (FEC—Equation (4)) and the coefficient of body weight gain per caloric intake (CWGCC), with the diet intake corresponding to 3.8 Kcal.g^−1^, as presented in Equation (5):

(4) $$FEC=\frac{Final\;Weight\;\left(g\right)-Initial\;Weight\;(g)}{Total\;Consumption\;(g)}$$

(5) $$CWGCC=\frac{Final\;Weight\;\left(g\right)-Initial\;Weight\;(g)}{Caloric\;value\;of\;the\;diet\;(kcal)}$$

### 2.4. Blood and Tissue Sampling

By the end of the experiment (PND100), all the animals fasted for 12 h for subsequent blood collection. The collection of 500 µL of blood was performed with the animal anesthetised with an intraperitoneal injection of 1.5 mL.kg^−1^ of xilazin (20 mg.mL^−1^, Rompun^®^, Bayer, Brazil) and 1.5 mL.kg^−1^ of ketamine (100 mg.mL^−1^; Dopalem^®^, Vetbrands, Brazil). After blood collection, animals were euthanised through a mixture of narcotic drugs (95 mg ketamine.kg^−1^ and 12 mg xilazin.kg^−1^) to induced overdosis. Subsequently, a laparotomy of the animal was performed to collect liver, brain, soleus muscle right and kidney right. The tissues were weighed and aliquoted into microtubes containing 1.5 mL of Tris-HCl buffer, pH 7.4, and subsequently stored at −80 °C.

The collected blood was refrigerated for subsequent complete blood count (hemoglobin and hematocrit; red blood cells (RBC) count; white blood cells (WBC) count and WBC differential count; and determination of the hematometric indices: mean corpuscular volume (MCV), mean corpuscular hemoglobin (MCH) and mean corpuscular hemoglobin concentration (MCHC) [35].

### 2.5. Antioxidant System and Liver Function Analyses

Tissue samples were centrifuged at 13,680× *g* at 4 °C for 12 min, and the post mitochondrial supernatant (PMS) was used for analysis. Protein was measured [36] and samples were normalized to determine the activity of the enzymes superoxide dismutase (SOD) [37], glutathione *S*-transferases (GST) [38], glutathione peroxidase (GPx) [39], and glutathione reductase (GR) [40]. Lipoperoxidation rates (LPO) were determined using the Buege and Aust method [41], and the activity of aspartate aminotransferase (AST) and alanine aminotransferase (ALT) using the correspondent kits from LabtestDiagnóstica S.A.

### 2.6. Statistical Analysis

The biometric variables (water consumption, body weight, naso-anal length, and Lee index) were analyzed by repeated measures ANOVA with Tukey HSD post hoc test. The total body weight gain, total food consumption, specific rate of body weight gain, feeding efficiency and body weight gain per caloric intake coefficients, blood parameters, and the enzymatic variables (SOD, GST, GR, GPx, LPO, ALT and AST) were checked for normality (Shapiro–Wilk test) and homoscedasticity (F test) and those that were in agreement with such assumptions were analyzed by T-test independent samples. When the assumptions were not in agreement, the Mann–Whitney U test was used.

The matrices of biometrics variables (body weight gain—WG, body weight gain rate—WGR, total consumption—TC, feeding efficiency—FE, body weight gain per caloric consumption—WGCC), blood parameters (hemoglobin, hematocrit, erythrocytes, leukocytes, stick neutrophil, segmented neutrophil, lymphocyte, monocyte, eosinophil, basophil, mean cell volume, mean cell hemoglobin and mean cell hemoglobin concentration), liver antioxidant system (SOD, GST, GR and GPx), muscle antioxidant system (SOD, GST, GR and GPx), kidney antioxidant system (SOD, GST, GR and GPx), brain antioxidant system (SOD, GST, GR and GPx), oxidative stress in tissues (muscle LPO, liver LPO, kidney LPO, brain LPO), and protein metabolism in liver (liver ALT and AST) were standardized (z-score). The matrices were then used for permutational multivariate analysis of variance (PERMANOVA) using the respective Euclidean distance, establishing as fixed factors the treatments (control, exposure). The pairwise comparisons for all pairs of levels of the fixed factor were performed by using the permutational MANOVA with Bonferroni correction method (“EcolUtils” package, adonis.pair function). The results were performed by biplot of principal coordinate analysis. All analyses were performed with a level of significance *p* = 0.05, using R [42].

## 3. Results

### 3.1. Biometric Variables

**Figure 2:**
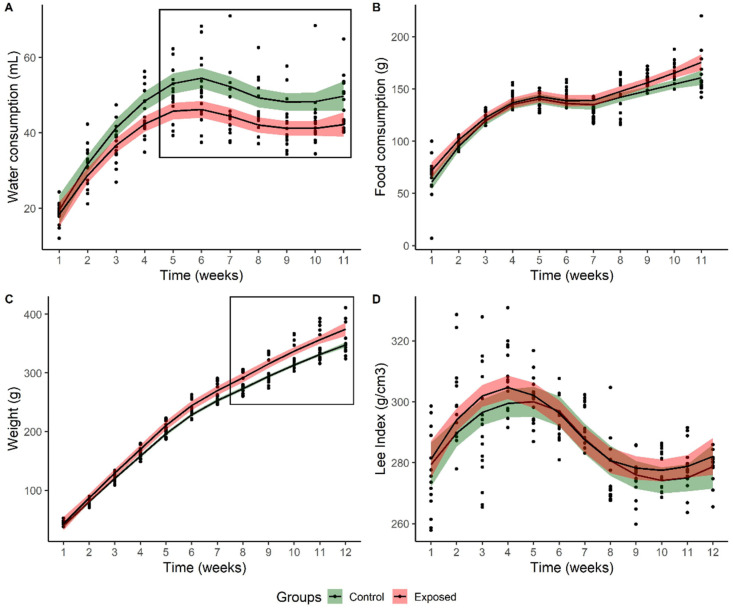
Variation in biometric parameters for Wistar rats exposed to no contaminated water (control) and water contaminated with DDD + DDE (0.002 mg.L^−1^ + 0.005 mg.L^−1^): (**A**) water consumption; (**B**) food consumption; (**C**) body weight; (**D**) Lee index. Boxes highlight significant differences between treatments.

Significantly lower water consumption was observed in the exposed group when compared to the control (F_1,140_ = 6.5, *p* = 0.023) despite a similar increase over time between groups (F_10,140_ = 1.5; *p* = 0.130, Figure 2A) as expected due to the organisms’ growth. The mean pesticide concentration consumed throughout the experiment was 5.8 ± 0.2 μg of DDD and 14.6 ± 0.6 μg of DDE. The food consumption was similar between groups (F_1,140_ = 3.9, *p* = 0.069) and constant throughout the experiment (F_10,140_ = 1.122; *p* = 0.350—Figure 2B). As for the mean body weight of animals, a significant increase was observed for the exposure group (F_1,154_ = 7.6; *p* = 0.015—Figure 2C). Finally, for the Lee index, no significant differences were found between groups throughout the experiment (F_1,154_ = 1.87; *p* = 0.193; Figure 2D).

The total body weight gain for the exposure group was significantly higher (t_14_ = −2.34, *p* = 0.046), despite the body weight gain rate not showing significant differences between groups (t_14_ = −0.12; *p* = 0.910). Total food consumption (t_14_ = −1.97; *p* = 0.069), feed efficiency (t_14_ = 1.45, *p* = 0.170) and body weight gain per caloric intake (t_14_ = 1.45, *p* = 0.170; Table 1) were similar between groups.

### 3.2. Blood Analyses

No significant differences were observed for all the blood parameters (Table 2), and neither eosinophil nor basophil were recorded in the studied animals.

### 3.3. Antioxidant System and Damage Tissue Analyses

When evaluating the liver tissue, GST (t_14_ = 2.15; *p* = 0.050) and ALT activities (t_14_ = 2.51; *p* = 0.026) were significantly higher in exposed organisms (Table 3).

The exposure to DDD and DDE significantly reduced SOD activity (t_14_ = 2.50; *p* = 0.026) and LPO rate in the soleus muscle (t_14_ = 4.27; *p* = 0.001). On the opposite side, GPx was significantly higher (t_14_ = 6.79; *p* < 0.0001), showing approximately double the activity in the control group (Table 3).

In renal tissue, SOD activity was significantly higher in the exposure group (t_14_ = 3.20; *p* = 0.006—Table 3), with the other biomarkers showing similar values.

In the brain, significant differences were observed only for GPx, with higher activity in the exposure group (t = 6.15; *p* < 0.0001; Table 3).

### 3.4. Integrative Analyses of the Antioxidant System and Tissue Damage

**Figure 3:**
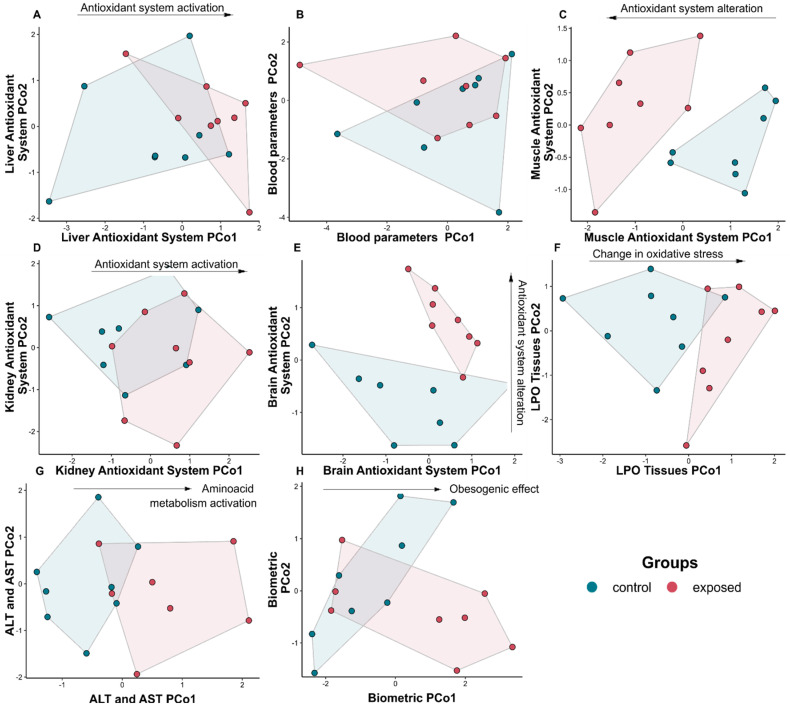
Principal coordinates analysis (PCoA) plot: (**A**) liver antioxidant system (SOD; GST; GPx and GR); (**B**) blood parameters (Hb, HT, RBC, WBC, SN, SEN, LY, MY, EP, BP, MCV, MCH and MCHC); (**C**) muscle antioxidant system; (**D**) kidney antioxidant system (SOD; GST; GPx and GR); (**E**) brain antioxidant system (SOD; GST; GPx and GR); (**F**) oxidative stress (LPO liver; LPO muscle; LPO kidney; LPO brain); (**G**) aminoacid metabolism (ALT and AST); (**H**) biometrics parameters (WG, WGR, TC, FE, WGCI).

The evaluation of the antioxidant system in the tissues was carried out in an integrated perspective, as enzymes depend on substrates formed throughout the process. These analyses did not show a significant increase in the activity of the antioxidant system in the liver of exposed animals (F_1,14_ = 2.50; *p* = 0.072—Figure 3A), but rather a discrete difference compared to the control group. There was an activation of the antioxidant system in the liver tissue, with a slight increase when compared to the control group, mainly represented by the activation of the GST enzymes, responsible for the biotransformation process. No significant differences were identified between the groups for the integrated blood parameters (F_1,14_ = 0.74; *p* = 0.641—Figure 3B). However, the other tissues (muscle, kidney and brain) of exposed animals showed significant changes. The soleus muscle of exposed animals showed inactivation of the antioxidant system, except for the higher GPx activity (F_1,14_ = 6.57; *p* = 0.002—Figure 3C). Higher activity of the antioxidant system was observed in kidney tissue (F_1,14_ = 2.73; *p* = 0.041—Figure 3D) and brain tissue of the exposed animals (F_1,14_ = 3.64; *p*= 0.003—Figure 3E).

The oxidative stress showed significant difference among LPO values (F_1,14_ = 4.11, *p* = 0.003, Figure 3F). This difference was a great evidence of oxidative stress in the kidney tissue and in the brain of animals exposed to DDD and DDE. A reduction in LPO values was observed in the soleus muscle. The low activity of the antioxidant system may indicate a deleterious effect from chronic exposure to trace concentrations of DDD and DDE, given the significant increase in ALT activity in animals exposed to such pesticides (F_1,14_ = 4.14; *p* = 0.018—Figure 3G). The biometric measures showed higher values among the contaminated animals (F_1,14_ = 2.50; *p* = 0.092—Figure 3H).

## 4. Discussion

In this work, the chronic exposure of Wistar rats to trace concentrations of DDT residues (DDD and DDE) and their possible impact on the hepatic, muscular, renal and nervous antioxidant systems, along with other biometric parameters of the animals (e.g., body weight, Lee index) were studied. The novelty and importance of the study herein are related to the use of environmentally relevant concentrations, as previously demonstrated by Fernandes et al. [43], in a populated area that is characterized by high rates of cancer diagnosis and the prevalence of higher rates of other diseases such as the palate cleavage [44]. Current Brazilian legislation does not allow any residual concentration of these pesticides in the environment due to their ban in 2009 according to Law N° 11,936, of May 14, 2009 [45]. Nonetheless, despite their ban, there are numerous records on the presence and persistence of DDT and its metabolites in soils and groundwater [11,28,29,30,43]. Although the negative impact of DDT and its metabolites has been reported in a review published by Agency for Toxic Substances and Disease Registry [46], the focus of research is mainly given to the study of high concentrations and their effects on public health [47].

**Figure 4:**
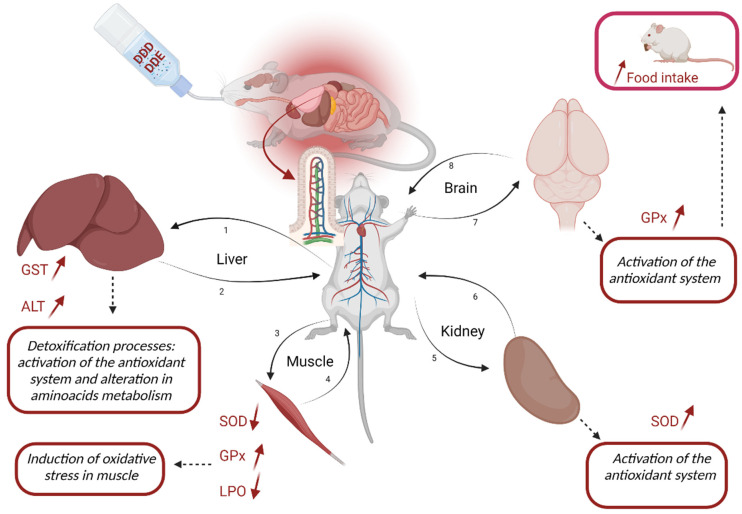
Overview of the results for Wistar rats chronically exposed to trace concentrations of DDD and DDE (0.002 mg.L^−1^ and 0.005 mg.L^−1^, respectively). The most significant absorption of DDD and DDE happens from the lymphatic way by the gastrointestinal tract, as mentioned by Jandacek et al. [54]. Afterward, DDD and DDE are distributed to tissues such as liver (1 and 2), muscles (3 and 4), kidney (5 and 6) and brain (7 and 8). The figure describes the changes in the enzymatic system and lipid peroxidation, and the consequences resulting from the metabolic process in each tissue. GST—Glutathione *S*-transferases; GPx—Glutathione peroxidase; SOD—Superoxide dismutase; LPO—Lipid peroxidation; ALT—Alanine aminotransferase. Created with BioRender.com.

The initial analysis of the antioxidant system, throughout the exposure period, barely showed any significant differences. As expected, the interpretation of these biomarkers, when evaluated separately, brings little information, as stated before, mainly due to their interconnection in cellular reactions. In addition, exposure to low concentrations like the ones used in this study, may give rise to minor, no significant variations in one or several biomarkers and are thus being neglected, even though the occurrence of oxidative stress processes is clear. Changes in a late sampling period have been previously observed in different studies [48,49,50]. Exposure to DDT and its metabolites in the neonatal period has negatively impacted future generations [51,52]. Skinner et al. [53] showed that parental exposures to DDT promoted health damage in the F3 generation, demonstrating a transgenerational epigenetic effect in both male and female animals. Thus, it is important to highlight that the nature and pattern of the observed changes are one of the key findings of this study. Although at earlier life stages, the animals appear to be in good health status, the negative impact of DDT metabolites may be observed at later life stages that will impact their life span (e.g., AST and ALT increased activities, cellular damaged shown by LPO rates). An overview scheme with the impact of DDT metabolites is presented below (Figure 4).

### 4.1. Liver Impact

As the primary detoxification organ, the observed change in liver function on amino acid metabolism in exposed animals (evidenced by increased ALT) may result from the stress caused by exposure to pesticides. Timoumi et al. [55] demonstrated that the liver could be the main target of pesticides, as it is responsible for the biotransformation of xenobiotics. Here, the pesticides are metabolized through cytochrome P450 and/or the glutathione enzymes before they reach more sensitive organs, such as the kidney and brain [56]. The attempt to make the molecules more water-soluble helps transport them out of the cell, giving rise to lower toxicity to the organism [57]. The detoxification process in the liver was not enough to eliminate the xenobiotics. This was evidenced by the changes in the antioxidant system of the tissues of the soleus muscle, the kidney and the brain.

### 4.2. Muscle Impact

As DDT metabolites reached the soleus muscle, a strong oxidative stress impact was observed as shown by SOD, GPx and LPO, as also previously reported by Chehade et al. [58]. The significant inhibition of SOD activity leads to a higher accumulation of superoxide anions in the muscle capable of causing rapid oxidative damage. Among these damages, superoxide is able to directly oxidize several biomolecules and inactivate enzymes with iron–sulfur centers, such as NADH dehydrogenase, fumarase, creatine kinase and calcineurin, leading to impaired muscular function [59]. Inhibition of SOD enzyme activity, in contrast to the increase in the GPx enzyme, may also result from the high production of hydrogen peroxide (H_2_O_2_) [60]. This molecule can diffuse through cell membranes, causing oxidative damage and promoting immediate cellular effects, such as structural changes in the cell and the recruitment of immune cells [59,61]. Loss of oxidative capacity in skeletal muscle seems to have a strong correlation to muscular mitochondrial dysfunction due to oxidative damage in mitochondrial proteins [58,62,63]. It is noteworthy to remember that SOD follows a bell-shaped pattern when, after a strong induction, it fails to continue the detoxification role and tends to inhibit levels lower than the basal ones. The increased activity of GPx denotes the presence of high oxidative stress and the need to reduce their levels, which are causing cellular damage to the cells, as shown by the significant inhibition of LPO rates. As oxidative stress increases, an increasing trend in LPO rates is also expected, leading to a decrease in cell viability. Nonetheless, when the impact is extreme, the decrease in LPO rates, as herein observed, is expected, as cells tend to die, and no LPO can be measured. This has been observed in previous studies such as Ferreira et al. [64]. This significant decrease in LPO rates relates to the increase in GPx activity that per se will confirm the similar levels of GST activity due to the consumption of reduced glutathione by GPx and the SOD inhibition.

### 4.3. Kidney Impact

Small changes in the antioxidant system of renal tissue were also observed in this study. Marouani et al. [65] pointed out that DDT was able to induce renal dysfunction (renal blood vessel congestion, glomerular atrophy, tubule degeneration in addition to extensive necrosis), being concomitant with an increased activity of GST and GPx, indicating adaptive responses of the organism to ROS. A human study identified a positive association between serum levels of DDE and the risk of chronic kidney disease, indicating that is possible that polymorphism of xenobiotics-metabolizing enzymes not only increased accumulation of pesticides, but also worsens kidney dysfunction, as shown by the decrease in estimated glomerular filtration rate [66]. Another factor that may indicate kidney tissue damage was the significant difference in water consumption between the groups. Animals exposed to residual concentrations of DDD and DDE consumed significantly smaller water volumes than animals in the control group. This result may be evidence of reduced organ function, with a consequent increase in blood pressure, which promotes a behavior of little thirst [67]. However, experimental tests with controlled groups need to be carried out to confirm this evidence.

### 4.4. Brain Impact

The activation of the antioxidant system in the brain, observed in the present study, is of great concern, considering that the central nervous system (CNS) is the most sensitive in the body and highly susceptible to chemicals [68]. Activation of the GPx enzyme in brain seems to indicate that the blood–brain barrier has been effectively overcome by pesticides. Gupta, Agarwa and Shukla [69] reported that rats’ blood–brain barrier can be highly vulnerable to certain types of pesticides. Even if the exposure is unique, the effects can be observed later on after the exposure has ceased. Researchers have long demonstrated that oxidative damage in different brain regions promotes the loss of cognitive and motor skills [70]. Thus, the activation of the antioxidant system presented here and the evidence of oxidative damage (increased lipid peroxidation—LPO) in animals chronically exposed to DDD and DDE can generate an accelerated decline in motor and cognitive performance. Previous studies have shown a possible association between exposure to DDT and its derivatives and a higher occurrence of cases with cognitive and mental deficits, together with higher risks of neurological diseases [15,18,19]. According to Richardson et al. [18], DDT and DDE can increase amyloid precursor protein levels, resulting in Aβ peptide. The production of this peptide is considered a precursor of Alzheimer’s disease since this product is neurotoxic and can lead to the formation of senile plaques and cell death [71].

### 4.5. Biometric Assay

It is worth mentioning that the homogenate of the analyzed brain tissue contained the hypothalamus. The hypothalamus is a critical brain region responsible for the organism’s homeostasis, controlling several functions, including appetite control, appetite-suppressant role and weight control [67]. The biometric results showed significant differences between the groups, with higher body weight observed in exposed animals. Those results may be related to the chronic exposure effect of DDD and DDE on the hypothalamic tissue. It is widely documented that DDT and its metabolites, mainly DDE, are responsible for increased adipose tissue accumulation, insulin resistance and dyslipidemia in humans [22] and rats [72]. Adipose tissue and insulin are important in the secretion and control of leptin secretion, a protein that acts on the hypothalamus to control food intake [73]. La Merrill et al. [74] demonstrated that perinatal exposure to DDT increased adiposity in young adult rats, reduced energy expenditure, and induced insulin resistance, which may be a result of impaired thermogenesis in exposed animals. In addition, recent studies have shown that pesticides can cause weight gain in rats chronically treated with low doses of pesticides and that this weight gain is also associated with changes in the gut microbiota of exposed animals [75,76].

In addition, higher body weight gain observed in the group exposed to DDD and DDE reflects changes in the appetite-suppressant role of the animals. According to Kim et al. [77], the induction of leptin expression, an adipokine responsible for appetite control and energy expenditure regulation, occurs when animals are exposed to DDT and DDE. DDE promotes changes in adipogenesis and influences adipocyte differentiation by modulating the levels of the CCAAT-alpha amplifier binding protein (C/EBPα) and the peroxisome proliferator-activated receptor gamma (PPARγ), along with lipid metabolism [77]. Obesogenic development may result from an accumulation of ectopic fat in skeletal muscle triggered by exposure to DDE [78]. In addition, the relationship of DDE with the modulation of PPARPy levels may prove the influence of the pesticide on mitochondrial biogenesis and ATP synthesis [79]. DDE could increase mitochondrial fatty acid oxidation and adipocyte mobilization, interfering with the role of energy generation [50]. Finally, there is already evidence in the literature that suggests a possible relationship between exposure to DDT and its metabolites, especially DDE, and adverse health effects such as obesity in children [80,81], together with the epigenetic transgenerational inheritance of obesity and many associated comorbidities.

## 5. Conclusions

The present study presents warning results regarding the chronic effects of animals exposed to trace concentrations of DDT metabolites (DDD and DDE). Despite DDT being definitively banned more than a decade ago (2009) in Brazil, and its use not allowed in agriculture for much longer (since 1985), trace concentrations of this chemical remain in soils and stand out among others within the environment. Although such trace concentrations are considered insignificant, this study shows they can directly impact Wistar rats’ health status. Considering that they can reach and contaminate water sources, such results are of great concern. For example, in rural areas, it can end by contaminating the water, the food and the air.

The combined effects of DDD and DDE are still unknown. This study is one of the first steps to better understand their impact on ecosystems and on human populations, highlighting the need for further studies.

## Figures and Tables

**Table 1:** Biometric evaluation results. Results expressed in mean ± standard error; * denotes significant difference from the control (*p* < 0.05).

Evaluation	Control	Exposure
Total body weight gain (g)	288.75 ± 3.30	313.375 ± 10.01 *
Total food consumption (g)	1436.00 ± 16.62	1504.62 ± 30.65
Body weight gain rate	6.65 ± 0.23	6.69 ± 0.21
Feeding efficiency	0.20 ± 0.003	0.21 ± 0.003
Body weight gain per caloric intake	0.053 ± 0.001	0.055 ± 0.001

**Table 2:** Blood parameters. Results expressed in mean ± standard error.

	Control	Exposure	*p*-Value
Hemoglobin concentration (g/dL)	13.79 ± 0.60	14.75 ± 0.76	0.057
Microhematocrit (%)	41.38 ± 1.81	44.25 ± 2.27	0.151
Red blood cells (10^6^/mm^3^)	8.21 ± 0.59	9.50 ± 0.61	0.1537
White blood cells (10^3^/mm^3^)	5.67 ± 0.50	5.86 ± 0.53	0.8015
Stick neutrophil (%)	0.63 ± 0.37	0.25 ± 0.16	0.5613
Segmented neutrophil (%)	18.88 ± 2.53	19.75 ± 2.50	0.8095
Lymphocyte (%)	79.75 ± 2.72	78.75 ± 2.40	0.7871
Monocyte (%)	0.75 ± 0.25	1.25 ± 0.41	0.317
Eosinophil (%)	0	0	-
Basophil (%)	0	0	-
Mean cell volume (fL)	52.34 ± 4.39	48.37 ± 4.15	0.5223
Mean cell hemoglobin (pg)	17.45 ± 1.46	16.12 ± 1.38	0.5223
Mean cell hemoglobin conc. (g/dL)	33.33 ± 0.00	33.33 ± 0.00	-

**Table 3:** Antioxidant system and tissue damage. Results expressed in mean ± standard error. * denotes a significant difference from the control (*p* < 005). **^£^** denotes the use of the Mann–Whitney U test.

Tissue	Analysis	Control	Exposure	*p*-Value
Liver	SOD	10.97 ± 1.69	12.53 ± 1.60	0.513
	GR	29.44 ± 2.30	34.20 ± 1.26	0.090
	GST	116.30 ± 10.76	142.11 ± 5.36 *	0.049
	GPx	5.79 ± 1.45	10.13 ± 2.52	0.158
	LPO	22.19 ± 1.93	19.87 ± 3.92	0.604
	ALT	23.64 ± 2.83	35.77 ± 4.02 *	0.026
	AST	84.89 ± 14.90	119.48 ± 16.69	0.145
Muscle	SOD	6.49 ± 0.37	5.14 ± 0.40 *	0.026
	GR	6.82 ± 0.36	6.55 ± 0.36	0.612
	GST	23.78 ± 0.49	22.35 ± 0.64	0.097
	GPX	83.07 ± 8.95	161.04 ± 7.20 *	<0.0001
	LPO	5.31 ± 0.64	1.77 ± 0.53 *	<0.001
Kidney	SOD	12.29 ± 0.78	16.63 ± 1.11 *	0.006
	GR	29.68 ± 2.35	28.62 ± 3.02	0.786
	GST	37.14 ± 3.83	41.55 ± 2.51	0.352
	GPx	38.28 ± 7.85	59.01 ± 9.85	0.122
	LPO	1.45 ± 0.56	2.48 ± 0.44	0.073 ^£^
Brain	SOD	9.72 ± 0.65	9.25 ± 0.59	0.603
	GR	14.39 ± 2.56	11.89 ± 1.13	0.396
	GST	31.19 ± 2.89	29.51 ± 0.62	0.878 ^£^
	GPx	120.23 ± 4.18	151.90 ± 3.00 *	<0.0001
	LPO	21.52 ± 2.66	28.66 ± 2.80	0.086

## Data Availability

Not applicable.
